# Refining the Immersive Technology Evaluation Measure for Virtual Reality Clinical Skills Among Native Arabic-Speaking Medical Students: Qualitative Study

**DOI:** 10.2196/95904

**Published:** 2026-09-28

**Authors:** Afaf Sulaiman Alblooshi, Falah Mohammed Almarzooqi, Taleb M Almansoori, Anmol Punn, Marwa Gaffar Alameen, Alexander Kieu, Faten Abdullah AlRadini

**Affiliations:** 1 Department of Medical Education, College of Medicine and Health Sciences, United Arab Emirates University, Al Ain, Abu Dhabi, United Arab Emirates; 2 Department of Family Medicine, College of Medicine and Health Sciences, United Arab Emirates University, Al Ain, Abu Dhabi, United Arab Emirates; 3 Department of Radiology, College of Medicine and Health Sciences, United Arab Emirates University, Sheikh Khalifa Street, Al Ain, Abu Dhabi, United Arab Emirates, 971 37137558; 4 Department of Family and Community Medicine, College of Medicine, Princess Nourah bint Abdulrahman University, Riyadh, Riyadh Region, Saudi Arabia

**Keywords:** interview, extended reality, virtual reality, immersive technology, interactive learning environments, medical education

## Abstract

**Background:**

Extended reality technologies, including virtual reality (VR), augmented reality, and mixed reality, are increasingly used in medical education to create immersive and interactive learning environments. As these modalities expand, validated instruments are needed to measure learners’ experiences accurately. The Immersive Technology Evaluation Measure (ITEM) is a multidomain questionnaire assessing immersion, motivation, cognitive load, usability, and debriefing. Although cognitive interviewing informed its original development, less is known about how ITEM questions function when used in a different linguistic and educational context.

**Objective:**

This study aimed to evaluate the clarity, comprehensibility, and response processes of ITEM among native Arabic-speaking medical students enrolled in an English-medium medical program. We also sought to identify linguistic, referential, and contextual sources of comprehension difficulty and use these findings to inform proposed item-level refinements.

**Methods:**

We conducted a qualitative cognitive interviewing study with 10 third-year and fourth-year medical students at United Arab Emirates University following VR-based clinical skills activities. Using concurrent think-aloud and verbal probing techniques, participants explained how they interpreted each ITEM question and arrived at their responses. Interview transcripts, recordings, and interviewer notes were independently reviewed using a descriptive, item-focused approach. Participant feedback was examined for recurring comprehension problems, and proposed item-level decisions were reviewed through research team consensus. Item-level saturation was reached after 8 interviews and confirmed with 2 additional interviews.

**Results:**

Participants identified comprehension or response-process problems in 47.5% (19/40) of ITEM questions. Difficulties occurred across all 5 domains and were most frequent in immersion (6/9, 66.7%) and usability (6/10, 60%), followed by debriefing (4/5, 80%), motivation (2/10, 20%), and cognitive load (1/6, 16.7%). Common difficulties involved nonspecific references such as “activity” and “technology,” unfamiliar or abstract terminology, negative wording, and unclear temporal or contextual framing. For example, “concern” was interpreted by some participants as worry rather than focus, and “mentally demanding” was interpreted in relation to mental health rather than cognitive effort. Cognitive interviewing informed different item-level decisions rather than a uniform revision: 11 problematic questions received proposed wording or contextual revisions, 2 received presentation-only modifications, and 6 were retained unchanged where clarification could introduce a meaning not clearly established in the original item. Six additional questions received limited contextual or terminology-standardizing changes for consistency. No items were removed, and the original 5-domain structure was retained.

**Conclusions:**

Cognitive interviewing identified response-process difficulties that were not apparent from the questionnaire wording alone and provided a systematic basis for determining when clarification was warranted and when the original wording should be preserved. The findings extend response-process evidence for ITEM and illustrate the value of examining established educational measures in new linguistic and educational settings. The proposed refinements provide a foundation for further cognitive testing and psychometric evaluation across immersive learning contexts.

## Introduction

Extended reality (XR) technologies, including virtual reality (VR), augmented reality (AR), and mixed reality (MR), are increasingly used in medical education to create immersive and interactive learning environments [1-4]. These modalities allow learners to engage with simulated clinical scenarios that support decision-making, procedural skills practice, and teamwork in a safe and engaging learning environment [5,6]. As immersive learning platforms expand in medical education, there is a parallel need for validated instruments that can reliably assess learners’ experiences and outcomes [7].

Several established questionnaires assess individual components of immersive learning; however, these tools are often applied independently and may fail to capture the broader, multidimensional nature of immersive learning experiences. For example, usability and technology acceptance are commonly assessed using the System Usability Scale (SUS) [8] and the technology acceptance model [9], presence and immersion using the iGroup Presence Questionnaire [10], perceived cognitive workload using the NASA Task Load Index (NASA-TLX) [11], and learner motivation using the intrinsic motivation inventory [12]. This limitation is particularly relevant in XR-based education, where learners’ perceptions may be shaped simultaneously by the level of immersion, system usability, motivation, cognitive demands, and opportunities for structured reflection. A comprehensive measure integrating complementary constructs can therefore support a more complete evaluation of learner experience in immersive technology-enhanced learning (TEL).

To address the need for a multidomain evaluation instrument, Jacobs et al [7] developed the Immersive Technology Evaluation Measure (ITEM), a 40-item self-report questionnaire designed to assess key dimensions of the learner’s experience in immersive environments. ITEM integrates 5 established measures and frameworks: the Adapted Immersion Experience Questionnaire (AIEQ) [13,14], abridged intrinsic motivation inventory (AIMI) [14,15], NASA-TLX [11], SUS [8], and PEARLS (Prompts for Engaging and Reflective Learning in Simulation) debriefing tool [16]. Together, these domains assess immersion, intrinsic motivation, cognitive load, system usability, and debriefing. Cognitive interviewing was included in the original development of ITEM to assess participants’ understanding of its questions [7]. However, cognitive evaluation during initial instrument development does not necessarily establish that questions will be interpreted similarly when an instrument is subsequently used in different linguistic, cultural, or educational contexts.

Questionnaires are widely used in medical education research to evaluate learners’ perceptions, attitudes, and experiences [17-20]. Notably, even survey items that appear straightforward to researchers can be interpreted differently by respondents, potentially introducing misunderstanding and response error [21-23]. Cognitive interviewing is a qualitative questionnaire-evaluation approach that examines how respondents understand questions and formulate responses [22-24]. The survey response process has commonly been conceptualized in terms of comprehension, retrieval of relevant information, judgment, and response formulation [24]. Cognitive interviewing uses techniques such as think-aloud (TA) and verbal probing (VP) to explore these processes and identify problems involving terminology, ambiguous wording, question complexity, and contextual interpretation [22,25-28]. It has also been used to examine whether questionnaire items function similarly across cultural and linguistic contexts [28-30].

This consideration is particularly relevant when respondents complete questionnaires in a language that is not their first language. Previous research has demonstrated that language proficiency and linguistic characteristics of survey questions, including complex syntax, ambiguous wording, and less familiar terminology, warrant consideration when questionnaires are administered across linguistic groups [31,32]. Such issues are relevant to medical education in the United Arab Emirates, where medical training may be delivered in English within a predominantly Arabic-speaking clinical and social environment [33]. Research involving Arabic-speaking university students has also demonstrated differences in knowledge and attitude scores when the same health-related questionnaire was administered in Arabic and English [34]. These findings do not imply inadequate English proficiency among learners studying in English-medium programs; rather, they highlight the potential value of examining how specific questionnaire terms and expressions are interpreted when the survey language differs from respondents’ first language.

Although cognitive interviewing was incorporated into the original development of ITEM [7], limited evidence is available regarding how its established English-language questions are interpreted by learners whose first language is not English when the instrument is administered without translation. Accordingly, this study aimed to evaluate the clarity, comprehensibility, and response processes of ITEM among native Arabic-speaking medical students enrolled in an English-medium medical program in the United Arab Emirates after VR-based clinical skills activities. Using cognitive interviewing with TA and VP techniques, we examined how participants interpreted individual ITEM questions and identified linguistic, referential, and contextual sources of comprehension difficulty. The findings were used to inform proposed linguistic or contextual clarifications where appropriate, while retaining the original wording when a modification could not be made confidently without potentially altering the intended meaning.

## Methods

### Study Design

This qualitative study used cognitive interviewing to evaluate the clarity, comprehensibility, and response processes of ITEM among native Arabic-speaking medical students enrolled in an English-medium medical program. We selected cognitive interviewing as an evidence-based questionnaire evaluation method to identify potential sources of misunderstanding and response error prior to broader administration of the instrument. Reporting was guided by the Cognitive Interviewing Reporting Framework (CIRF) [28] (Checklist 1).

### Setting

We conducted the study in September 2025 at the Simulation Center (iSTAR), College of Medicine and Health Sciences, United Arab Emirates University (UAEU), in the United Arab Emirates. The undergraduate medical curriculum is delivered in English, while Arabic is the first language of the enrolled United Arab Emirates–national student population. The UAEU medical program is a 6-year curriculum with premedical, preclinical, and clinical phases; we recruited participants from years 3 and 4 because these preclinical years represent a transition into structured clinical skills training supported by simulation-based education.

### Ethical Considerations

The Social Sciences Ethics Committee at the UAEU approved the study (ERSC_2025_8044). Participation was voluntary. Participants received no financial compensation or other incentives for participation. We obtained written informed consent electronically prior to participation using REDCap, a secure, web-based data capture platform designed to support research data collection and management [35,36]. Participants consented to audiovisual recording for transcription and analysis and were informed that they could decline to answer any question or withdraw at any time without penalty. We stored data securely with access restricted to the research team. We removed identifying information during transcript processing and analyzed anonymized transcripts to protect participant privacy and confidentiality.

### Instrument

ITEM is a 40-item multidomain questionnaire designed to capture user experience in immersive TEL environments [7] (Multimedia Appendix 1). Participants completed the original English-language ITEM wording during the cognitive interviews. The questionnaire integrates 5 domains derived from established frameworks and validated measures: AIEQ [13,14] measures immersion and system fidelity, AIMI [14,15] assesses learner motivation and perceived educational value, NASA-TLX [11] evaluates cognitive and physical load associated with task performance, SUS [8] assesses confidence and perceived accessibility of the technology, and PEARLS [16] explores reflective learning and debriefing processes.

Proposed wording decisions arising from the cognitive interviews are presented separately and were not administered in a second round of cognitive testing.

### Participants and Recruitment

Participants were undergraduate medical students in years 3 and 4 of the UAEU undergraduate medical program who had completed the preliminary VR clinical skills activity. Twenty students participated in the preliminary activity and were informed verbally about the cognitive interview study; all were subsequently invited by email. Ten students volunteered to participate. Interviews were intended to continue until no new item-level comprehension issues emerged rather than to achieve a predetermined fixed sample size. Saturation was identified after 8 interviews through ongoing review and team discussion, and 2 additional interviews were conducted to confirm that no new relevant item-level issues emerged. All 10 participants were United Arab Emirates nationals and native Arabic speakers. English-language proficiency and prior language of schooling were not formally assessed or collected.

### VR Experience (Context for Questionnaire Evaluation)

Prior to the cognitive interviews, participants completed a curriculum-aligned VR clinical skills session. Participants also completed a presession demographic and XR-background questionnaire in REDCap (Multimedia Appendix 2). Facilitators first provided a technical orientation explaining the VR equipment, headset controls, and activity sequence. All participants used the same VR platform and followed the same instructional process, although the clinical module differed according to year-level curricular content. Year 3 students were randomly allocated to 1 of 2 modules (vital signs, n=3; and intravenous cannula insertion, n=1), and year 4 students completed a nasogastric tube insertion module (n=6). Each student completed the guided procedure individually. After the VR activity, participants provided verbal reflections organized broadly around strengths, weaknesses, opportunities, and threats. This activity was intended to elicit general feedback on usability, engagement, and instructional value and did not constitute a formal structured simulation debrief or use the PEARLS framework. The study did not compare cognitive interview findings across VR modules. Figure 1 provides an overview of the study process.

**Figure 1.**
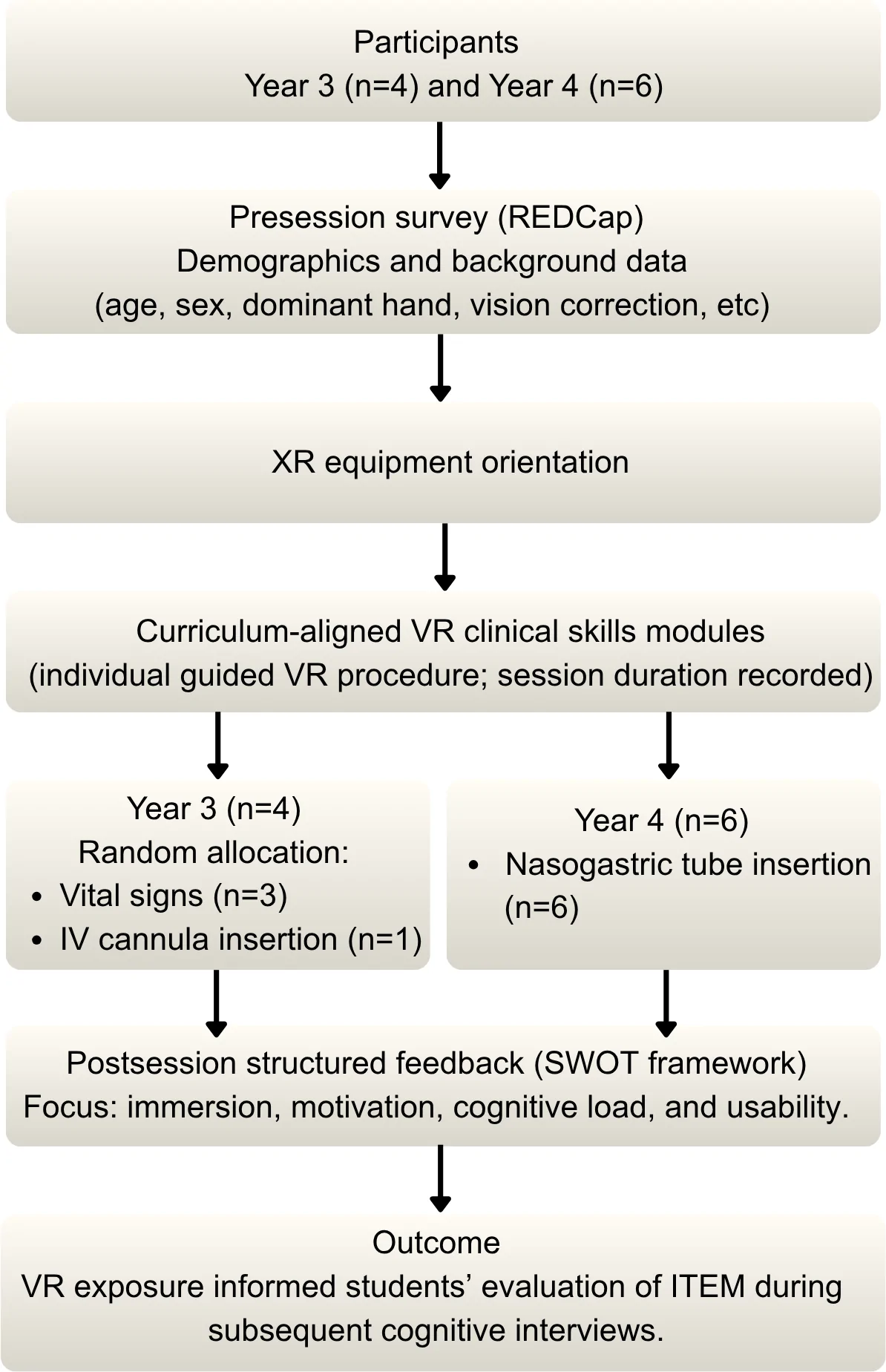
Workflow of the structured virtual reality (VR) clinical skills session and subsequent cognitive interview evaluation of the Immersive Technology Evaluation Measure (ITEM). IV: intravenous; SWOT: strengths, weaknesses, opportunities, and threats.

### Cognitive Interview Procedure

Following the VR learning sessions, year 3 (n=4) and year 4 (n=6) students participated in cognitive interviews. Two faculty members (FMA and AK) conducted the interviews online via Microsoft Teams (version 25240.1603.3956.3103). Both interviewers were experienced in medical education and familiar with the use of cognitive interviewing procedures. Neither interviewer participated in the students’ VR teaching sessions or assessment. With participant permission, we recorded interviews and transcribed them verbatim. Interviews were scheduled for 30 minutes and lasted 20 to 45 minutes depending on participant engagement. Participants were asked to keep their cameras on to support observation of nonverbal cues such as facial expressions or hesitation. Interviewers used a combination of concurrent and retrospective cognitive interviewing techniques [22]: TA (concurrent) [26], whereby participants were prompted to think aloud while reading each survey item aloud, verbalizing their thoughts, interpretations, and reasoning as they answered the questions; VP (concurrent) [27], whereby interviewers used a combination of prescripted and spontaneous probes to clarify interpretation, meaning, and response selection; and retrospective debriefing [22], whereby, after completing the questionnaire, participants were asked to reflect on overall clarity, confusing terms, and any culturally unfamiliar wording.

Interviewers used a combination of prescripted probes and spontaneous follow-up probes when participants hesitated, requested clarification, or provided an interpretation requiring further exploration.

### Data Management and Item-Level Analysis

We transcribed recordings using Otter AI (version 3.93.0; Otter.ai, Inc) and manually checked each transcript against the recording for accuracy. Identifying information was removed prior to analysis. Two researchers (AP and MGA) independently reviewed all transcripts, recordings, and interviewer notes. Participant comments were organized in Microsoft Excel by ITEM question, together with observations of hesitation, requests for clarification, and alternative interpretations.

The analysis was descriptive and item-focused rather than a formal thematic analysis. For each ITEM question, the researchers first summarized the specific comprehension or response-process difficulties raised by participants. These item-level observations were then compared across questions to identify recurring types of difficulty. Recurring problems were organized into descriptive categories comprising ambiguous referents, unfamiliar or abstract terminology, negative wording, temporal ambiguity, and contextual relevance. These categories were used to organize and summarize the item-level findings; they were not intended to represent a formal qualitative coding framework or thematic analysis. An item was considered problematic when participants expressed confusion, requested clarification, hesitated because of uncertainty about meaning, or provided differing interpretations that indicated potential ambiguity. Item-level findings, participant-reported problems, modification decisions, and the rationale for retaining or revising each question were documented in an item-level audit (Multimedia Appendix 3).

AP and MGA compared their item-level assessments and assignment of descriptive problem categories after independent review and did not identify substantive disagreements. The wider research team subsequently reviewed the participant comments and proposed item-level decisions. Any differences during team discussion were resolved through consensus. No specialized qualitative analysis software or interrater agreement statistic was used.

Proposed wording changes were kept deliberately conservative. Where participant feedback indicated a comprehension problem that could be clarified without intentionally changing the item meaning, the team considered a brief linguistic or contextual clarification. Where the intended scope of an item could not be established confidently from the original wording or source measure, the original wording was retained rather than imposing a new interpretation. Multimedia Appendix 4 presents the proposed wording and presentation modifications. A native English-speaking language expert from the United States reviewed the proposed wording for linguistic clarity. The proposed wording was not subjected to a second round of cognitive interviews in this study.

### Rigor and Reflexivity

To support consistency and transparency, 2 researchers independently reviewed the interview transcripts, recordings, and interviewer notes, and item-level findings were subsequently discussed with the wider research team. Neither cognitive interviewer participated in the students’ VR teaching sessions or assessment. Because several members of the research team were involved in immersive TEL implementation, the team considered how their familiarity with VR and ITEM could influence interpretation of participant comments. Proposed item-level decisions were therefore discussed collectively and grounded in participants’ statements, observed comprehension difficulties, and interviewer notes, with any differences in interpretation resolved through consensus.

## Results

### Participants

Ten undergraduate medical students participated in the cognitive interviews (n=4 in year 3 and n=6 in year 4). The mean age was 20 (SD 0.5; range 20‐21) years for year 3 students and 21 (SD 0.9; range 20‐22) years for year 4 students. Nine participants were female and 1 was male. All participants were United Arab Emirates nationals and native Arabic speakers enrolled in an English-medium medical program. All participants completed a VR-based clinical skills session prior to the interviews.

### Cognitive Interview Duration and Item-Level Saturation

Interviews were scheduled for 30 minutes and lasted 20 to 45 minutes, with a mean duration of approximately 33 minutes. Item-level saturation was reached after 8 interviews, with no new comprehension problems identified. Two additional interviews were conducted to confirm saturation, and no new relevant item-level issues emerged.

### Overview of Item-Level Findings

Across the 40 questions of ITEM, participants identified 47.5% (19/40) as presenting a comprehension or response-process problem. Problems occurred across all 5 domains: immersion (6/9, 66.7%), usability (6/10, 60%), debriefing (4/5, 80%), motivation (2/10, 20%), and cognitive load (1/6, 16.7%). The most common difficulties involved ambiguous or nonspecific referents, unfamiliar or abstract terminology, negative wording, temporal ambiguity, and contextual relevance.

Of the 19 participant-identified problematic questions, 11 (57.9%) received proposed wording or contextual revisions, 2 (10.5%) received presentation-only modifications without alteration of the original wording, and 6 (31.6%) were retained in their original wording. An additional 6 questions that were not independently identified as problematic received limited contextual or terminology-standardizing changes for consistency. The complete item-level findings and decision rationale are provided in Multimedia Appendix 3. Illustrative examples of revisions made following participant feedback are presented in Table 1.

**Table 1.** Illustrative cognitive interview findings and proposed item-level decisions for the Immersive Technology Evaluation Measure (ITEM)^a^.

Original question (Q)	Problem identified	Representative participant comment	Item-level decision
Section 1: immersion			
Q1: I was interested in seeing how the activity would progress	Ambiguous reference to “activity”	“Which activity are they talking about here?” “What activity do you mean? So, the VR in general, like the whole study, just my experience with the VR? So, I think specifying the activity would be good.”	Proposed contextual clarification: “I was interested in seeing how the activity (simulation session using VR) would progress.”
Q6: At the time, the activity was my only concern	“Concern” interpreted as worry rather than focus	“Why concern? It was not a concern. It was something nice.”	Proposed plain-language clarification: “At the time, the activity was my only concern (focus).”
Q7: I wanted to learn more about the outcome following the activity	Unclear “outcome” referent.	“Outcome of what?” “Like, would it be applied or introduced in our university? Is that what they mean?”	Original wording retained. Participants interpreted “outcome” differently, and the intended referent could not be established confidently without imposing a new interpretation.
Section 2: motivation			
Q13: This activity did not hold my attention at all	Negative wording and response directionality	“What do they mean by this question?” “Okay? No, I was like immersed in it, but I am still confused actually with the DID NOT HOLD.”	No wording revision. “DID NOT” was capitalized as a typographic emphasis to draw attention to the negative construction: “This activity DID NOT hold my attention at all.”
Section 3: cognitive load			
Q20: How mentally demanding was the task?	Misinterpretation of the phrase “mentally demanding”	“How much I had focused into the task, or did I understand it wrong?” “When I read mentally demanding, I thought about mental health and not in a thinking way.”	Proposed plain-language clarification: “How mentally demanding (needs a lot of focus) was the task?”
Section 4: usability			
Q27: I found the technology unnecessarily complex	Difficulty interpreting “unnecessarily complex”	“So, it is not complex, or it is complex?” “This word. Do they mean I found the technology complex?”	Proposed clarification and contextual specification: “I found the VR technology unnecessarily complex (more complicated than necessary).”
Q29: I think that I would need the support of a technical person to be able to use this technology	Unclear temporal scope (initial vs ongoing support)	“Do you mean every time I use it, or only the first time?” “I think the first time, I think the first time we need someone, but after that, I don’t think we need someone. So, I don’t know what to put my answer.”	Original wording retained. The source item does not specify a temporal frame; therefore, no clarification was introduced.

a Examples are illustrative; complete item-level findings and decision rationales for all 40 ITEM questions are provided in Multimedia Appendix 3. Proposed wording and presentation modifications were not cognitively retested in this study.

### Patterns of Comprehension Difficulty

A recurring issue across domains was the use of nonspecific references such as “activity” and “technology.” Participants variously interpreted these as referring to the VR software, the clinical procedure, or the overall simulation session. Where appropriate, proposed contextual clarifications specified the VR activity or VR technology to reduce uncertainty regarding the referent.

Participants also reported difficulty with abstract or unfamiliar terms. For example, some were uncertain about the meaning of “immersion,” and one participant interpreted “mentally demanding” as referring to mental health rather than the amount of cognitive effort required. Negatively worded statements also created response uncertainty. In response, typographic emphasis was proposed for “DID NOT” in question 13 without altering the wording of the item.

Not all identified problems resulted in wording changes. For question 7, participants differed in their interpretation of “outcome,” and for question 29, they questioned whether technical support referred to initial or ongoing use. Because these meanings could not be established confidently from the original wording, the original questions were retained rather than introducing a clarification that could alter their intended scope.

### Debriefing Domain

Participants identified comprehension difficulties in 4 of the 5 debriefing-domain questions. However, participants had not undergone a formal structured postactivity debrief; the brief strengths, weaknesses, opportunities, and threats–style reflection following the VR activity was not based on the PEARLS framework. Consequently, these difficulties could not be attributed confidently to questionnaire wording alone and may have reflected limited contextual applicability. The original wording of all debriefing-domain questions was therefore retained.

### General Feedback

Across interviews, participants described some questions as broad, formal, or unfamiliar and indicated that brief contextual cues or plain-language explanations could support comprehension of selected terms. Most identified issues concerned wording or contextual interpretation rather than the overall structure of ITEM. No participant feedback indicated a need to remove an ITEM domain or change the questionnaire’s overall multidomain structure.

## Discussion

### Principal Findings

This study used cognitive interviewing to evaluate the clarity, comprehensibility, and response processes of the ITEM among native Arabic-speaking medical students enrolled in an English-medium medical program. Participants identified comprehension or response-process difficulties across all 5 ITEM domains, including ambiguous referents, unfamiliar or abstract terminology, negative wording, temporal ambiguity, and contextual relevance.

The findings demonstrated that response-process difficulties can emerge when an established English-language instrument is used in a different linguistic and educational context, even when learners study in English. Importantly, cognitive interviewing informed not only where clarification might be useful but also where retaining the original wording was more appropriate because the intended meaning could not be established confidently. The resulting item-level decisions therefore included proposed linguistic clarifications, contextual specifications, presentation changes, and deliberate retention of original wording. These findings provide response-process evidence for ITEM in this context but do not demonstrate that the proposed revisions themselves improved interpretability.

### Comparison With Prior Work

Our findings are consistent with cognitive interviewing literature showing that respondents may interpret apparently straightforward questionnaire items differently according to wording, context, and linguistic experience [22,23,28,29]. Cross-cultural cognitive interviewing research similarly emphasizes that equivalent interpretation should not be assumed solely because respondents can complete an instrument in the language in which it was developed [29,30].

Research on survey response across linguistic groups also supports consideration of language proficiency and linguistic complexity when questionnaires are administered across languages [31,32]. In an Arabic-speaking context, differences have been reported when the same health-related questionnaire was administered in Arabic and English [34]. Although these studies differ from the present study in design and population, they support examining response processes when questionnaire language differs from respondents’ first language. The present study extends this work by examining an established English-language immersive technology measure without translating it, reflecting how ITEM would be used in an English-medium medical education setting in the United Arab Emirates [33].

### Domain-Specific and Contextual Findings

Comprehension problems occurred across all ITEM domains but were most frequent in the immersion and usability domains. Several of these questions contained abstract terminology or broad referents such as “activity,” “technology,” and “outcome,” which participants interpreted in different ways. Where clarification could be introduced without intentionally changing the item scope, brief contextual or plain-language explanations were proposed.

Findings from the debriefing domain required separate interpretation. Participants identified difficulties with 4 of the 5 debriefing questions; however, they had not undergone a formal structured postactivity debrief. The brief strengths, weaknesses, opportunities, and threats–style reflection was not a PEARLS-based debriefing process. Because PEARLS was developed specifically for structured simulation debriefing [7,16], these difficulties may have reflected limited contextual applicability rather than wording alone. The original wording of all debriefing questions was therefore retained.

The cognitive interview findings also highlighted the importance of avoiding overcorrection. For question 7, the meaning of “outcome” remained uncertain, and for question 29, the original item did not specify whether technical support referred to initial or ongoing use. Rather than introducing a meaning not clearly established in the source items, we retained the original wording. This conservative approach recognizes that cognitive interviewing can identify ambiguity without necessarily providing sufficient evidence to redefine an item [28,29], and even apparently minor questionnaire adaptations should be considered carefully because they may affect item functioning [37].

### Implications for Medical Education and Instrument Use

These findings support cognitive interviewing as a practical method for examining response-process evidence when established educational measures are applied beyond their original development context [22,23,28,29]. For ITEM, this study provides an item-level account of where participants encountered uncertainty and documents the rationale for proposed linguistic, contextual, or presentation modifications. Because the proposed wording was not cognitively retested, these modifications should be considered provisional rather than validated improvements.

The addition of “VR” to selected proposed clarifications does not make ITEM a VR-only instrument. ITEM was developed for immersive TEL and was originally evaluated in both VR and AR contexts [7]. In this study, “VR” was added only where participants found the generic term “technology” insufficiently specific. In other immersive settings, the original technology-neutral wording could be retained or the relevant modality, such as AR or MR, could be specified where needed.

More broadly, studying medicine in English does not necessarily mean that all English-language questionnaire expressions will be interpreted uniformly. Response-process evaluation may therefore complement psychometric testing when established instruments are used in different linguistic or educational settings.

### Limitations

This study has several limitations. First, the proposed wording was not cognitively retested; therefore, the study identifies response-process difficulties and proposes potential refinements but cannot establish that the revisions resolved the identified comprehension problems. Second, English-language proficiency and prior language of schooling were not formally assessed, so the findings cannot determine whether the observed comprehension difficulties varied according to individual language proficiency or educational language background. Third, only 1 male student participated, which limited our ability to examine whether interpretation differed by sex. The sample was also drawn from a single institution. These characteristics constrain subgroup comparisons and broader generalizability; however, the purpose of cognitive interviewing was to identify and characterize item-level comprehension problems rather than estimate their prevalence in the wider student population. Item-level saturation was reached after 8 interviews and confirmed with 2 additional interviews, supporting the adequacy of the sample for the study’s primary cognitive interviewing objective. Fourth, participants did not undergo a formal structured debrief; therefore, difficulties with the PEARLS-derived items may reflect limited contextual applicability rather than questionnaire wording alone. Finally, interviews were conducted online via Microsoft Teams. In some cases, screen sharing disabled participants’ cameras, a common constraint when using tablets or iPads, which limited observation of nonverbal cues such as facial expressions and hesitations. Online interviewing may also reduce opportunities for rapport building compared with in-person interviews, which could have affected the depth of spontaneous verbalizations during the TA process.

### Future Directions

Future research should cognitively retest the proposed item-level modifications to determine whether they resolve the identified problems without introducing new interpretations. Subsequent studies should evaluate reliability, construct validity, and measurement performance in larger and more diverse samples, including learners with different linguistic backgrounds and levels of English proficiency. Further investigations across VR, AR, and MR settings and in activities involving formal structured debriefing would also help determine when modality-specific contextual wording is appropriate.

### Conclusions

Cognitive interviewing identified linguistic, referential, and contextual response-process difficulties in several ITEM questions among native Arabic-speaking medical students enrolled in an English-medium medical program. The findings informed proposed item-level clarifications while also identifying questions for which retaining the original wording was more appropriate because further modification could impose an interpretation not clearly established in the source item.

These findings provide additional response-process evidence for ITEM in a different linguistic and educational context but do not establish that the proposed wording changes improve interpretability. Further cognitive testing and subsequent psychometric evaluation are needed before the proposed refinements can be considered confirmed.

## Supplementary material

10.2196/95904 Multimedia Appendix 1 Immersive Technology Evaluation Measure questionnaire administered during cognitive interviews (original wording).

10.2196/95904 Multimedia Appendix 2 Presession participant questionnaire capturing demographic and background information.

10.2196/95904 Multimedia Appendix 3 Immersive Technology Evaluation Measure item-level cognitive interview audit.

10.2196/95904 Multimedia Appendix 4 Proposed modified Immersive Technology Evaluation Measure following cognitive interview review.

10.2196/95904 Checklist 1 Cognitive Interviewing Reporting Framework (CIRF) checklist.

## Abbreviations

AIEQ: Adapted Immersion Experience Questionnaire
AIMI: abridged intrinsic motivation inventory
AR: augmented reality
ITEM: Immersive Technology Evaluation Measure
MR: mixed reality
NASA-TLX: NASA Task Load Index
PEARLS: Prompts for Engaging and Reflective Learning in Simulation
SUS: System Usability Scale
TA: think-aloud
TEL: technology-enhanced learning
UAEU: United Arab Emirates University
VP: verbal probing
VR: virtual reality
XR: extended reality
