# Supplementary material for: Refining the Immersive Technology Evaluation Measure for Virtual Reality Clinical Skills Among Native Arabic-Speaking Medical Students: Qualitative Study
Source: JMIR Med Educ. 2026 Sep 28;12:e95904. doi: 10.2196/95904 (PMC13618990; doi:10.2196/95904)
Supplement: Supplementary file 2 [file mededu-v12-e95904-s002.docx]

| **Multimedia Appendix 3.** Presession participant questionnaire capturing demographic and background information. | | |
| --- | --- | --- |
|  | **Question** | **Response** |
| 1. | Name |  |
| 2. | Student ID |  |
| 3. | Age |  |
| 4. | Gender | ☐ Male  ☐ Female |
| 5. | Year of Study | ☐ 3rd Year  ☐ 4th Year |
| 6. | Height (in cm) |  |
| 7. | Do you wear glasses/lenses? | ☐ Yes  ☐ No |
| 8. | Specify the power of your glasses/lenses for the Right eye |  |
| 9. | Specify the power of your glasses/lenses for the Left eye |  |
| 10. | Dominant hand | ☐ Right-handed  ☐ Left-handed  ☐ Ambidextrous |
| 11. | Do you have any of the following problems? (select all that apply) | ☐ None  ☐ Inner ear problems (including hearing impairment) ☐ Dizziness  ☐ Middle ear problems  ☐ Other |
| 12. | If other, please specify |  |
| 13. | Do you experience light-headedness (feel like you might pass-out/ faint) within the last 3 month? | ☐ Yes  ☐ No |
| 14. | Do you suffer from motion sickness? | ☐ Yes  ☐ No |
| 15. | Have you previously participated in ANY simulation-based learning? | ☐ Yes  ☐ No |
| 16. | Do you have experience with Augmented Reality (AR), Virtual Reality (VR), or Mixed Reality (MR)? | ☐ Yes  ☐ No |
| 17. | What is your comfort level with using this technology? | ☐ Not Comfortable at All  ☐ Slightly Comfortable  ☐ Moderately Comfortable  ☐ Very Comfortable  ☐ Extremely Comfortable |
| 18. | Have you ever used AR, VR, or MR technology for educational purposes? | ☐ Yes  ☐ No |
| 19. | Frequency of Use of AR, VR, MR | ☐ Never  ☐ Rarely  ☐ Occasionally  ☐ Frequently  ☐ Very Frequently |
