# Supplementary material for: Refining the Immersive Technology Evaluation Measure for Virtual Reality Clinical Skills Among Native Arabic-Speaking Medical Students: Qualitative Study
Source: JMIR Med Educ. 2026 Sep 28;12:e95904. doi: 10.2196/95904 (PMC13618990; doi:10.2196/95904)
Supplement: Supplementary file 3 [file mededu-v12-e95904-s004.docx]

| **Multimedia Appendix 2.** Proposed Modified Immersive Technology Evaluation Measure (ITEM) Following Cognitive Interview Review  **Modified wording is indicated by an asterisk (*), bold text, and underlining in the revised items* | | | | | | |
| --- | --- | --- | --- | --- | --- | --- |
| Please rank your response below to the statements. | | | | | | |
| **IMMERSION** | | | | | | |
|  | | Strongly disagree | Somewhat disagree | Neither agree nor disagree | Somewhat agree | Strongly agree |
| 1. | I was interested in seeing how the activity **(simulation session using VR)*** would progress | ☐ | ☐ | ☐ | ☐ | ☐ |
| 2. | I enjoyed the visuals **(graphics)*** of the activity | ☐ | ☐ | ☐ | ☐ | ☐ |
| 3. | **(**It was as if**)*** I could interact with the simulated environment as if I was in the real world | ☐ | ☐ | ☐ | ☐ | ☐ |
| 4. | I was unaware of what was happening around me, external to the simulated environment | ☐ | ☐ | ☐ | ☐ | ☐ |
| 5. | I felt detached from the outside world | ☐ | ☐ | ☐ | ☐ | ☐ |
| 6. | At the time, the activity was my only concern **(focus)*** | ☐ | ☐ | ☐ | ☐ | ☐ |
| 7. | I wanted to learn more about the outcome following the activity | ☐ | ☐ | ☐ | ☐ | ☐ |
| 8. | To me it felt like only a short amount of time had passed | ☐ | ☐ | ☐ | ☐ | ☐ |
| 9. | How Immersed did you feel? **(how realistic was the experience)*** | 0 1 2 3 4 5 6 7 8 9 10 | | | | |
| Your immersion score  (1=low immersion, 5=moderately immersed, 10=very high immersion) | |  | | | | |
| **MOTIVATION** | | | | | | |
|  | | Strongly disagree | Somewhat disagree | Neither agree nor disagree | Somewhat agree | Strongly agree |
| 10. | I enjoyed doing this activity **(simulation session using VR)*** very much | ☐ | ☐ | ☐ | ☐ | ☐ |
| 11. | I would describe this activity as interesting | ☐ | ☐ | ☐ | ☐ | ☐ |
| 12. | While I was doing this activity, I was thinking about how much I enjoyed it | ☐ | ☐ | ☐ | ☐ | ☐ |
| 13. | This activity **DID NOT*** hold my attention at all | ☐ | ☐ | ☐ | ☐ | ☐ |
| 14. | I believe this activity could be of value **(beneficial)*** to me | ☐ | ☐ | ☐ | ☐ | ☐ |
| 15. | I would be willing to do this again because it has value **(benefit)*** to me | ☐ | ☐ | ☐ | ☐ | ☐ |
| 16. | I think I can learn from this activity | ☐ | ☐ | ☐ | ☐ | ☐ |
| 17. | I think I am good at this activity | ☐ | ☐ | ☐ | ☐ | ☐ |
| 18. | I am satisfied with my performance at this task | ☐ | ☐ | ☐ | ☐ | ☐ |
| 19. | After working at this task for a while, I felt competent | ☐ | ☐ | ☐ | ☐ | ☐ |
| **COGNITIVE LOAD** | | | | | | |
|  | | Very low | Slightly low | Average | Slightly high | Very high |
| 20. | How mentally demanding **(needs a lot of focus)*** was the task? | ☐ | ☐ | ☐ | ☐ | ☐ |
| 21. | How physically demanding was the task? | ☐ | ☐ | ☐ | ☐ | ☐ |
| 22. | How hurried or rushed was the pace of the task? | ☐ | ☐ | ☐ | ☐ | ☐ |
| 23. | How successful were you in accomplishing what you were asked to do? | ☐ | ☐ | ☐ | ☐ | ☐ |
| 24. | How hard did you have to work to accomplish your level of performance? | ☐ | ☐ | ☐ | ☐ | ☐ |
| 25. | How insecure, discouraged, irritated, stressed, and annoyed were you? | ☐ | ☐ | ☐ | ☐ | ☐ |
| **USABILITY** | | | | | | |
|  | | Strongly disagree | Somewhat disagree | Neither agree nor disagree | Somewhat agree | Strongly agree |
| 26. | I would like to use this **VR*** technology frequently | ☐ | ☐ | ☐ | ☐ | ☐ |
| 27. | I found the **VR*** technology unnecessarily complex **(more complicated than necessary)*** | ☐ | ☐ | ☐ | ☐ | ☐ |
| 28. | I thought the **VR*** technology was easy to use | ☐ | ☐ | ☐ | ☐ | ☐ |
| 29. | I think that I would need the support of a technical person to be able to use this technology | ☐ | ☐ | ☐ | ☐ | ☐ |
| 30. | I found the various functions of the **VR*** technology were well integrated **(worked well together)*** | ☐ | ☐ | ☐ | ☐ | ☐ |
| 31. | I thought there was too much inconsistency with this **VR*** technology **(it did not work the same every time)*** | ☐ | ☐ | ☐ | ☐ | ☐ |
| 32. | I would imagine that most people would learn to use this **VR*** technology quickly | ☐ | ☐ | ☐ | ☐ | ☐ |
| 33. | I found the **VR*** technology awkward to use | ☐ | ☐ | ☐ | ☐ | ☐ |
| 34. | I felt very confident using the **VR*** technology | ☐ | ☐ | ☐ | ☐ | ☐ |
| 35. | I needed to learn a lot of things before I could get going with **(start using)*** this **VR*** technology | ☐ | ☐ | ☐ | ☐ | ☐ |
| **DEBRIEF** | | | | | | |
|  | | Strongly disagree | Somewhat disagree | Neither agree nor disagree | Somewhat agree | Strongly agree |
| 36. | Did this feel like a safe learning environment? | ☐ | ☐ | ☐ | ☐ | ☐ |
| 37. | Did you explore your feelings? | ☐ | ☐ | ☐ | ☐ | ☐ |
| 38. | Was the narrative background and context of the simulated world made clear? | ☐ | ☐ | ☐ | ☐ | ☐ |
| 39. | Was one or more of your performance domains explored? For example, knowledge, procedural skills, or behaviours. | ☐ | ☐ | ☐ | ☐ | ☐ |
| 40. | Were key learning points to take-away identified? | ☐ | ☐ | ☐ | ☐ | ☐ |
| Note: Proposed wording and presentation modifications were informed by cognitive interview findings and research team review and were not cognitively retested in this study. | | | | | | |
