# Supplementary material for: Refining the Immersive Technology Evaluation Measure for Virtual Reality Clinical Skills Among Native Arabic-Speaking Medical Students: Qualitative Study
Source: JMIR Med Educ. 2026 Sep 28;12:e95904. doi: 10.2196/95904 (PMC13618990; doi:10.2196/95904)
Supplement: Supplementary file 4 [file mededu-v12-e95904-s005.docx]

**Multimedia Appendix 4**

**Cognitive Interviewing Reporting Framework (CIRF) Checklist**

**Checklist source:** Boeije H, Willis G. The Cognitive Interviewing Reporting Framework (CIRF): Towards the harmonization of cognitive testing reports. Methodology. 2013;9(3):87-95. doi:10.1027/1614-2241/a000075.

| **No.** | **CIRF guide question / description** | **Reported?** | **Where reported** |
| --- | --- | --- | --- |
| **1. Research objectives** | | | |
| 1a | What are the aims of the study? | **Yes** | Introduction (Study Objectives) |
| 1b | What is the context that gave rise to pretesting the instrument? | **Yes** | Introduction (Background and Study Objectives) |
| 1c | What is the theoretical perspective for the cognitive interviewing study? | **Yes** | Introduction (Response Process and Cognitive Interviewing) |
| **2. Research design** | | | |
| 2a | What was the basis for each feature of the design? | **Yes** | Methods (Study Design; Cognitive Interview Procedure) |
| **3. Ethics** | | | |
| 3a | Was the study approved by an ethics committee or IRB, including consent procedures? | **Yes** | Methods (Ethics) |
| 3b | How was the research project introduced to settings and participants? | **Yes** | Methods (Participants and Recruitment) |
| 3c | How were people motivated to participate? | **Yes** | Methods (Ethics; Participants and Recruitment) |
| 3d | How was confidentiality and anonymity of participants/sources protected? | **Yes** | Methods (Ethics; Data Management and Item-Level Analysis) |
| **4. Participant selection** | | | |
| 4a | What are participant details with respect to demographics and other project-specific items of information? | **Yes** | Methods (Participants and Recruitment); Results (Participants) |
| 4b | Did the selection of participants satisfy the study objectives? | **Yes** | Methods (Participants and Recruitment); Discussion (Limitations) |
| **5. Data collection** | | | |
| 5a | Who conducted the interviews and how many interviewers were involved? | **Yes** | Methods (Cognitive Interview Procedure) |
| 5b | How were the interviewers trained? | **Yes** | Methods (Cognitive Interview Procedure) |
| 5c | Were sessions recorded and, if so, was audio or video used? | **Yes** | Methods (Cognitive Interview Procedure; Data Management and Item-Level Analysis) |
| 5d | Were notes taken and how were they employed? | **Yes** | Methods (Data Management and Item-Level Analysis) |
| 5e | What type of verbal reporting method was employed (think-aloud, probing, or combinations)? | **Yes** | Methods (Cognitive Interview Procedure) |
| 5f | Was the interview protocol adjusted during the research process and, if so, how? | **No** | Methods (Cognitive Interview Procedure; no formal protocol adjustment reported) |
| 5g | Was saturation achieved? | **Yes** | Methods (Participants and Recruitment); Results (Cognitive Interview Duration and Item-Level Saturation) |
| **6. Data analysis** | | | |
| 6a | How were raw data transformed into categories representing problem areas and solutions? | **Yes** | Methods (Data Management and Item-Level Analysis) |
| 6b | What software programs were used? | **Yes** | Methods (Data Management and Item-Level Analysis) |
| 6c | How was reliability considered, including repetition of parts of the analysis by more than one researcher? | **Yes** | Methods (Data Management and Item-Level Analysis; Rigor and Reflexivity) |
| 6d | How did researchers work together and how were systematic analysis procedures encouraged? | **Yes** | Methods (Data Management and Item-Level Analysis; Rigor and Reflexivity) |
| 6e | Were there efforts to seek diverse observations (eg, triangulation)? | **Yes** | Methods (Data Management and Item-Level Analysis; Rigor and Reflexivity) |
| 6f | Was quantitative evidence used to supplement qualitative evidence? | **Yes** | Results (Overview of Item-Level Findings) |
| **7. Findings** | | | |
| 7a | What was observed concerning participant behavior with respect to each evaluated item? | **Yes** | Results; Table 1; Multimedia Appendix 5 |
| 7b | To what extent did results differ as a function of participant characteristics, behaviors, or status? | **No** | Results (findings reported at the overall item level; subgroup comparisons were not conducted) |
| **8. Conclusions, implications, and discussion** | | | |
| 8a | If possible, include a copy of the modified questions if one was produced as a product of testing. | **Yes** | Multimedia Appendix 2; Multimedia Appendix 5 |
| 8b | How do findings and solutions relate to previous evidence? | **Yes** | Discussion (Comparison With Prior Work; Domain-Specific and Contextual Findings) |
| **9. Strengths and limitations of the study** | | | |
| 9a | What were relevant a priori expectations or previous experiences? | **Yes** | Methods (Rigor and Reflexivity); Introduction |
| 9b | What are the implications of findings for generalization to the wider population or applicability to other settings? | **Yes** | Discussion (Implications; Limitations; Future Directions) |
| 9c | What is the study's contribution to methodological development and future practice? | **Yes** | Discussion (Implications for Medical Education and Instrument Use; Future Directions) |
| **10. Report format** | | | |
| 10a | Include main study documents relevant for independent inspection as appendices or online materials. | **Yes** | Multimedia Appendices 1-5 |
